# Association of Typical versus Atypical Antipsychotics with Symptoms and Quality of Life in Schizophrenia

**DOI:** 10.1371/journal.pone.0037087

**Published:** 2012-05-16

**Authors:** Koichiro Fujimaki, Terumichi Takahashi, Shigeru Morinobu

**Affiliations:** 1 Faculty of Health and Welfare, Prefectural University of Hiroshima, Hiroshima, Japan; 2 Mihara Hospital, Hiroshima, Japan; 3 Programs for Biomedical Research, Division of Frontier Medical Science, Department of Psychiatry and Neurosciences, Graduate School of Biomedical Sciences, Hiroshima University, Hiroshima, Japan; The University of Queensland, Australia

## Abstract

**Background:**

Several reports on patients with chronic schizophrenia suggest that atypical versus typical antipsychotics are expected to lead to better quality of life (QOL) and cognitive function. Our aim was to examine the association of chronic treatment with typical or atypical antipsychotics with cognitive function, psychiatric symptoms, QOL, and drug-induced extrapyramidal symptoms in long-hospitalized patients with schizophrenia.

**Methodology and Principal Findings:**

The Hasegawa Dementia Scale-Revised (HDS-R), Brief Psychiatric Rating Scale (BPRS), the Schizophrenia Quality of Life Scale, translated into Japanese (JSQLS), and the Drug-Induced Extrapyramidal Symptoms Scale (DIEPSS) were used to evaluate cognitive function, psychiatric symptoms, QOL, and drug-induced extrapyramidal symptoms. We examined the correlation between the dose of antipsychotics and each measure derived from these psychometric tests. The student *t*-test was used to compare scores obtained from psychometric tests between patients receiving typical and atypical antipsychotics. Results showed significant correlations between chlorpromazine (CPZ)-equivalent doses of typical antipsychotics and atypical antipsychotics, and the total BPRS score and BPRS subscale scores for positive symptoms. CPZ-equivalent doses of typical antipsychotics were correlated with the JSQLS subscale score for dysfunction of psycho-social activity and DIEPSS score. Furthermore, the total BPRS scores, BPRS subscale score for positive symptoms, the JSQLS subscale score for dysfunction of psycho-social activity, and the DIEPSS score were significantly higher in patients receiving typical antipsychotics than atypical antipsychotics.

**Conclusion and Significance:**

These findings suggest that long-term administration of typical antipsychotics has an unfavorable association with feelings of difficulties mixing in social situations in patients with chronic schizophrenia.

## Introduction

The use of atypical antipsychotics in the treatment of schizophrenia was introduced in Japan in 1996. Risperidone was approved in June 1996, followed by perospirone and quetiapine in February 2001, olanzapine in June 2001, aripiprazole in January 2006, and blonanserin in January 2008. After approval, risperidone was often used in addition to typical antipsychotics. Ongoing experience revealed the efficacy of risperidone as monotherapy, and this drug is currently one of the first-line agents used in the treatment of schizophrenia [1]. Although atypical antipsychotics have been recognized as first-line drugs in the treatment of schizophrenia in Japan, in actual clinical practice, typical antipsychotics are still prescribed to long-hospitalized patients with schizophrenia [2], [3]. Switching from typical antipsychotics to atypical antipsychotics usually takes place in Japan when an exacerbation of psychiatric symptoms is observed. In other words, therapeutic agents are rarely changed if no problematic behaviors are observed. Therefore, patients with chronic schizophrenia tend to receive the same drug regimen for many years [4]. Long-term administration of the same typical antipsychotic also makes it difficult to taper anticholinergics that are used to alleviate adverse effects induced by antipsychotics (e.g., extrapyramidal symptoms) [5]. The combination of typical antipsychotics and anticholinergics is often found in long-hospitalized patients with schizophrenia in Japan. However, growing evidence demonstrates the unfavorable effects of typical antipsychotics and/or anticholinergics on cognitive function [6]–[9]. Several reports focusing on inpatients with chronic schizophrenia suggest that switching from typical to atypical antipsychotics improves cognitive dysfunction [10]–[13].

In recent years, quality of life (QOL) has become an important issue. Social and occupational impairments have long been recognized as core features of schizophrenia affecting social interactions, vocational and instrumental functioning skills, self-care, and recreation [14]. Some cross-sectional studies of chronic schizophrenia have suggested that psychopathology might be more strongly correlated with community functioning than cognition [15], [16]. Various clinical factors related to QOL have been reported. Several studies have suggested that depressed mood may be the most important determinant of QOL [17]–[22]. Other studies have reported that positive symptoms [23] or akathisia symptoms, as well as the total severity of psychopathology [24], help predict subjective QOL. Regarding the influence of antipsychotics on QOL, Mortimer *et al.* reported that QOL is genuinely superior with atypical agents even allowing for the confounding effects of differential prescribing habits [25]. Furthermore, Ritsner *et al.* reported that both self-reported and rater-observed QOL measures indicated superiority of atypical over typical antipsychotic agents [26]. In the present study, we focused on whether chronic administration of antipsychotics influenced subjective QOL.

With these concerns in mind, we evaluated the association of chronic administration of antipsychotics with cognitive function, psychiatric symptoms, QOL, and drug-induced extrapyramidal symptoms in long-hospitalized patients with chronic schizophrenia and compared these measures between patients receiving typical and atypical antipsychotics.

## Methods

### Subjects

In total, 144 patients with schizophrenia participated in this study. Participants were chosen from patients who were hospitalized from 2000 to 2009. For patients who had been hospitalized two times or more, data from the latest evaluation were used. Duration of hospitalization represents the duration of hospital stay at the time of the assessments. There was one patient in the typical antipsychotic only group who had been hospitalized two times and one patient in the atypical antipsychotic only group who had been hospitalized three times. The minimum duration of hospitalization was 2.0 years in the typical antipsychotic only group, and 1.8 years in the atypical antipsychotic only group. Therefore, hospitalization data suggest that all patients had a long hospital stay. All participants met the criteria for schizophrenia according to the ICD-10 diagnostic classification. No patient had any other psychiatric disorder. The antipsychotic regimen had not been changed for at least 6 months in any subject before recruitment. All patients received one antipsychotic for at least 6 months before recruitment. All patients were taking typical antipsychotics or atypical antipsychotics. Typical antipsychotics included bromperidol (6–36 mg/day, *n* = 4), chlorpromazine (12.5–450 mg/day, *n* = 11), haloperidol (0.75–33 mg/day, *n* = 21), levomepromazine (5–200 mg/day, *n* = 12), and propericiazine (30–60 mg/day, *n* = 4). Atypical antipsychotics included aripiprazole (6–30 mg/day, *n* = 8), olanzapine (2.5–20 mg/day, *n* = 27), perospirone (4–48 mg/day, *n* = 5), quetiapine (10–750 mg/day, *n* = 20), and risperidone (0.5–12 mg/day, *n* = 32). Patients were divided into two groups: one group (n = 52) was receiving typical antipsychotics and another group (n = 92) was receiving atypical antipsychotics. In this analysis, only patients not receiving anticholinergics for at least 6 months before the assessment day were enrolled to eliminate the influence of anticholinergic drugs. In the group receiving typical antipsychotics only, 13 (25.0%) patients received one benzodiazepine that was added to one antipsychotic, 2 (3.85%) patients were on two benzodiazepines, 2 (3.85%) patients were on three benzodiazepines, and 1 (1.92%) patient was on four benzodiazepines. According to the definition in this study that polypharmacy was the concomitant use of two or more psychotropics, 18 participants (34.62%) were receiving psychotropic polypharmacy. In the group receiving atypical antipsychotics only, 27 (29.35%) patients received a single benzodiazepine that was added to a single antipsychotic, 5 (5.43%) patients were on two benzodiazepines, 1 (1.09%) patient was on three benzodiazepines, and 1 (1.09%) patient was on four benzodiazepines. Thirty-four participants (36.96%) were receiving psychotropic polypharmacy. In the group receiving typical antipsychotics only, typical antipsychotic medication had not been switched to atypical antipsychotic medication since the onset of schizophrenia. In the group receiving atypical antipsychotics only, atypical antipsychotic medication had not been switched to typical antipsychotic medication since typical antipsychotic medication was switched to atypical antipsychotic medication after 1996 in cases with disease onset before 1996. In cases with disease onset after 1996, atypical antipsychotic medication had not been switched to typical antipsychotic medication.

The study was approved by the ethics committee of Mihara Hospital. The content of the study and ethical considerations related to subjects were explained to subjects, and written informed consent to participate in the study was obtained.

### Variables assessed

Variables including amount of medication, age, age at disease onset, duration of disease, duration of hospitalization, years of education, duration of antipsychotic medication, neurocognitive function, psychotic symptoms, and drug-induced extrapyramidal symptoms were assessed by clinicians. QOL was determined using a rater-administered self-assessment scale. All variables were assessed on the same day. Each variable was assessed a single time. Gender, age, age at disease onset, duration of disease, duration of hospitalization, years of education, and duration of the antipsychotic medication were assessed based on medical charts.

All patients were taking typical or atypical antipsychotics. We used the chlorpromazine (CPZ) equivalent (mg) to determine the amount of typical and atypical antipsychotics each patient was receiving [27].

Neurocognitive functioning was measured using nine items on the Revised Hasegawa's Dementia Scale (HDS-R). The total score ranges from 0 to 30, with higher scores indicating better neurocognitive function [28].

The Brief Psychiatry Rating Scale (BPRS) was used to evaluate the severity of psychotic symptoms [29]. Each of 18 BPRS items was scored on a 7-point scale (0 to 6), with higher scores indicating more severe symptoms. Except for one item (mannerisms and posturing), each of 17 items was classified into four categories. The four categories were positive symptoms, negative symptoms, psychological discomfort, and resistance [30]. Positive symptoms were represented by the total score of five items (conceptual disorganization, suspiciousness, hallucinatory behavior, unusual thought content, disorientation). In the same way, negative symptoms, psychological discomfort, and resistance each were represented by the total score of three items (emotional withdrawal, motor retardation, blunted affect), five items (somatic concern, anxiety, guilt feelings, tension, depressive mood), and four items (grandiosity, hostility, uncooperativeness, excitement), respectively. The total BPRS score is the sum of scores for all items. All raters attended a formal training course on the use of the BPRS. Five training sessions of 3 hours each were conducted, including an explanation of the instrument's characteristics and rules, exercises on BPRS application and ratings, and formal testing of interrater reliability using videotaped interviews. During the course of data collection, three refresher meetings were held, discussing problems and confirming interrater reliability.

The primary dependent measure of interest was assessed using the Schizophrenia Quality of Life Scale, translated into Japanese (JSQLS). JSQLS is a rater-administered scale that assesses overall QOL and functioning using 30 items rated from 0 to 4, with higher scores reflecting worse QOL. This scale yields measures on three subscales that address 1) dysfunction of psycho-social activity, 2) dysfunction of motivation and energy, 3) level of symptoms and side effects. This scale shows high sensitivity to both changes and treatment effects and moderate-to-high correlations with other measures of QOL, and has been shown to have substantial sensitivity to subtle changes and treatment effects [31]. Each scale score is transformed to have a range from 0 (the best status as measured on JSQLS) to 100 (the worst status as measured JSQLS), with each scale calculated as follows: the scale score (*SS*) equals the total of raw scores of each item in the scale (*RS*_tot_), divided by the maximum possible raw score of all items in the scale (*RS*_max_), all multiplied by 100: *SS* = (*RS*_tot_/*RS*_max_)×100 [32].

The Drug-Induced Extrapyramidal Symptoms Scale (DIEPSS) was used to evaluate and exclude the effects of drug-induced extrapyramidal symptoms that could affect the severity of symptoms in schizophrenia patients. This scale is based on nine items rated from 0 to 4, with higher scores indicating more severe symptoms [33].

### Analytical methods

Partial correlations among scores on the psychometric tests (HDS-R, BPRS, JSQLS, DIEPSS) and the CPZ-equivalent doses of typical and atypical antipsychotics were calculated by Pearson linear correlation coefficients, with correlation coefficients at a level of 1% indicating significance. Partial correlation was performed to investigate the relationship between CPZ-equivalent dose and each psychometric test scores while controlling for age, age at disease onset, duration of disease, duration of hospitalization, years of education, duration of antipsychotic medication, and the other psychometric tests individually. The purpose was to find a unique variance between the two variables while eliminating the variance from a third variable.

Partial correlation analysis was applied to indicate the CPZ-equivalent doses of typical and atypical antipsychotics when age, age at disease onset, duration of disease, duration of hospitalization, years of education, duration of the antipsychotic medication, HDS-R score, total BPRS score, BPRS subscale score, JSQLS subscale score, and DIEPSS score were partialled out.

Correlations among clinical variables (age, age at disease onset, duration of disease, duration of hospitalization, years of education, and duration of antipsychotic medication) and psychometric test scores (HDS-R, BPRS, JSQLS, DIEPSS) were calculated by Pearson linear correlation coefficients, with correlation coefficients at a level of 1% indicating significance.

The student *t*-test was used to compare scores obtained from CPZ-equivalent dose of antipsychotics, age, age at disease onset, duration of disease, duration of hospitalization, years of education, duration of the antipsychotic medication and psychometric tests between patients receiving typical and atypical antipsychotics. Differences were considered significant at *P*<0.01. Statistical analyses were performed using PASW Statistics 18.0 software (SPSS Japan Inc., Tokyo, Japan).

## Results

Characteristics of subjects are shown in Table 1. All subjects were Japanese. The mean age of the 52 patients in groups receiving typical was 54.9 years, and 32.6% were male. The mean age of the 92 patients in groups receiving atypical was 59.1 years, and 39.1% were male. Variables assessed included CPZ-equivalent dose of antipsychotics, age, age at disease onset, duration of disease, duration of hospitalization, years of education, and duration of the antipsychotic medication. We compared each variable assessed between groups receiving typical and atypical antipsychotics. No significant differences in CPZ-equivalent dose of antipsychotics, age, age at disease onset, duration of disease, duration of hospitalization, years of education, and duration of the antipsychotic medication were seen between groups.

**Table 1:** Patients' characteristics (mean ± SD).

	Typical antipsychotic only group	Atypical antipsychotic only group	Between group p value
**No. of patients**	52	92	
**Gender % male**	32.6	39.1	
**CPZ equivalent dose of antipsychotics (mg/day)**	561.8±266.2	577.9±282.7	0.432
**Age (years)**	54.9±13.1	59.1±16.9	0.102
**Age at disease onset (years)**	24.3±8.1	24.6±8.2	0.901
**Duration of disease (years)**	18.8±10.1	21.7±12.1	0.864
**Duration of hospitalization (years)**	10.1±8.9 (minimum duration: 2.0, maximum duration: 23.1)	8.3±6.6 (minimum duration: 1.8, maximum duration: 28.3)	0.518
**Years of education (years)**	10.5±2.2	11.2±2.1	0.440
**Duration of the antipsychotic medication (years)**	17.9±9.6	19.1±12.8	0.223

Scores on evaluation scales are mean values (standard deviation).

Student's *t*-test.

CPZ, chlorpromazine.

The correlations between each psychometric test score and CPZ-equivalent doses of typical antipsychotics and atypical antipsychotics are shown in Tables 2 and 3. There were no significant relationships between the equivalent doses of typical or atypical antipsychotics and total HDS-R score. Significant positive correlations were found between CPZ-equivalent doses of typical and atypical antipsychotics and total BPRS score as well as the BPRS subscale score for positive symptoms. The CPZ-equivalent doses of typical but not atypical antipsychotics showed a significant positive correlation with the JSQLS subscale score for the dysfunction of psycho-social activity. The CPZ-equivalent doses of typical antipsychotics but not atypical antipsychotics were correlated with the DIEPSS score.

**Table 2:** Relationship between typical antipsychotic dose and each evaluation scale (correlation coefficient).

Evaluation scales		Partial correlation coefficients with changes after adjusting for variables between typical antipsychotics and each evaluation scale															
	*r* before adjusting for variables	Age	Onset age	Duration of disease	Duration of hospitalization	Years of education	Duration of the antipsychotic medication	HDS-R	BPRS total	BPRS positive symptoms	BPRS negative symptoms	BPRS psychological discomfort	BPRS resistance	JSQLS dysfunction of psycho- social activity	JSQLS dysfunction of motivation and energy	JSQLS level of symptoms and side-effect	DIEPSS
**HDS-R**	0.108	0.111	0.118	0.115	0.102	0.107	0.129		0.126	0.133	0.124	0.108	0.133	0.109	0.093	0.101	0.133
**BPRS total**	0.273 *	0.254 *	0.288 *	0.263 *	0.246 *	0.271 *	0.279 *	0.260 *						0.256 *	0.271*	0.271 *	0.222 *
**BPRS positive symptoms**	0.245 *	0.223 *	0.268 *	0.230 *	0.239 *	0.244 *	0.240 *	0.236 *						0.230 *	0.245 *	0.243 *	0.191 *
**BPRS negative symptoms**	0.117	0.032	0.109	0.038	0.081	0.104	0.028	−0.001						−0.057	−0.050	−0.011	0.016
**BPRS psychological discomfort**	0.089	0.112	0.131	0.122	0.137	0.113	0.092	0.091						0.085	0.068	0.109	0.105
**BPRS resistance**	−0.040	−0.023	−0.095	−0.011	−0.055	−0.033	−0.033	−0.044						−0.104	−0.100	−0.071	−0.012
**JSQLS dysfunction of psycho-social activity**	0.164 *	0.168 *	0.176*	0.162 *	0.180*	0.188*	0.167 *	0.163 *	0.166 *	0.168 *	0.162 *	0.161 *	0.160 *				0.168 *
**JSQLS dysfunction of motivation and energy**	0.053	0.093	0.102	0.096	0.072	0.090	0.047	0.050	0.067	0.084	0.093	0.011	0.128				0.093
**JSQLS level of symptoms and side-effect**	0.032	0.048	0.082	0.053	0.052	0.074	0.041	0.031	0.022	0.027	0.045	−0.074	0.079				0.104
**DIEPSS**	0.168 *	0.186 *	0.211*	0.172 *	0.206*	0.184*	0.210 *	0.202 *	0.183 *	0.191 *	0.162 *	0.159 *	0.163 *	0.212 *	0.213 *	0.166 *	

* Correlation is significant at the 1% level (two-sided).

BPRS, Brief Psychiatry Rating Scale; DIEPSS, The Drug-Induced Extrapyramidal Symptoms Scale; HDS-R, Revised Hasegawa's dementia scale; JSQLS, the Schizophrenia Quality of Life Scale, a Japanese version.

**Table 3:** Relationship between atypical antipsychotic dose and each evaluation scale (correlation coefficient).

Evaluation scales		Partial correlation coefficients with changes after adjusting for variables between atypical antipsychotics and each evaluation scale															
	*r* before adjusting for variables	Age	Onset age	Duration of disease	Duration of hospitalization	Years of education	Duration of the antipsychotic medication	HDS-R	BPRS total	BPRS positive symptoms	BPRS negative symptoms	BPRS psychological discomfort	BPRS resistance	JSQLS dysfunction of psycho- social activity	JSQLS dysfunction of motivation and energy	JSQLS level of symptoms and side-effect	DIEPSS
**HDS-R**	0.096	0.069	0.115	0.064	0.101	0.086	0.071		0.098	0.105	0.074	0.084	0.075	0.058	0.061	0.120	0.113
**BPRS total**	0.173 *	0.197 *	0.167 *	0.162 *	0.168 *	0.171 *	0.185 *	0.171 *						0.160 *	0.163 *	0.257 *	0.169 *
**BPRS positive symptoms**	0.253 *	0.229 *	0.218 *	0.233 *	0.228 *	0.265 *	0.273 *	0.255 *						0.247 *	0.254 *	0.229 *	0.255 *
**BPRS negative symptoms**	0.117	0.122	0.107	0.120	0.115	0.081	0.112	0.097						−0.010	0.012	−0.011	0.115
**BPRS psychological discomfort**	0.094	0.088	0.077	0.092	0.084	0.077	0.081	0.080						−0.025	−0.002	0.124	0.095
**BPRS resistance**	0.142	0.139	0.087	0.148	0.128	0.099	0.128	0.127						0.052	0.063	−0.062	0.145
**JSQLS dysfunction of psycho-social activity**	0.152	0.117	0.112	0.075	0.085	0.106	0.098	0.126	0.137	0.140	0.094	0.118	0.069				0.152
**JSQLS dysfunction of motivation and energy**	0.053	0.093	0.056	0.096	0.096	0.108	0.102	0.050	0.067	0.084	0.093	0.011	0.128				0.093
**JSQLS level of symptoms and side-effect**	0.136	0.134	0.109	0.128	0.129	0.106	0.131	0.122	0.119	0.112	0.076	0.103	0.066				0.135
**DIEPSS**	0.041	0.073	0.040	0.056	0.058	0.048	0.057	0.066	−0.020	−0.053	0.030	0.035	0.046	0.046	0.048	0.151	

* Correlation is significant at the 1% level (two-sided).

BPRS, Brief Psychiatry Rating Scale; DIEPSS, The Drug-Induced Extrapyramidal Symptoms Scale; HDS-R, Revised Hasegawa's dementia scale; JSQLS, the Schizophrenia Quality of Life Scale, a Japanese version.

The correlations among clinical variables and psychometric test scores are shown in Tables 4 and 5. There were significant positive correlations among age, duration of disease, duration of hospitalization, and duration of the antipsychotic medication in two groups. In addition, significant positive correlations were found between the DIEPSS score and the JSQLS subscale for dysfunction of psycho-social activity, and between BPRS negative symptoms or psychological discomfort or resistance and all JSQLS subscales, respectively.

**Table 4:** Relationship among clinical variables and psychometric test scores in groups receiving typical antipsychotics (correlation coefficient).

Variables assessed	Age	Onset age	Duration of disease	Duration of hospitalization	Years of education	Duration of the antipsychotic medication	HDS-R	BPRS total	BPRS positive symptoms	BPRS negative symptoms	BPRS psychological discomfort	BPRS resistance	JSQLS dysfunction of psycho- social activity	JSQLS dysfunction of motivation and energy	JSQLS level of symptoms and side-effect	DIEPSS
**Age**	1.0	0.221	0.364 *	0.471 *	−0.124	0.278 *	−0.130	−0.095	−0.075	−0.119	−0.102	−0.122	−0.140	−0.127	−0.105	0.104
**Onset age**	0.221	1.0	−0.144	−0.151	−0.095	−0.158	0.052	0.042	0.047	0.045	0.102	0.154	0.096	−0.014	0.139	−0.042
**Duration of disease**	0.364 *	−0.144	1.0	0.512 *	−0.110	0.672 *	−0.106	0.14	0.097	−0.026	0.016	−0.120	−0.134	−0.009	−0.072	0.127
**Duration of hospitalization**	0.471 *	−0.151	0.512 *	1.0	−0.014	0.488 *	−0.139	−0.047	−0.077	−0.106	−0.133	−0.129	−0.160	−0.082	−0.117	0.110
**Years of education**	−0.124	−0.095	−0.110	−0.014	1.0	−0.088	0.145	0.141	0.147	−0.035	0.060	0.111	0.100	−0.101	0.048	0.003
**Duration of the antipsychotic medication**	0.278 *	−0.158	0.672 *	0.488 *	−0.088	1.0	−0.095	0.131	0.078	−0.029	0.019	−0.126	−0.117	−0.016	−0.054	−0.021
**HDS-R**	−0.130	0.052	−0.106	−0.139	0.145	−0.095	1.0	−0.115	−0.017	0.121	0.151	0.138	0.121	0.137	0.140	−0.140
**BPRS total**	−0.095	0.042	0.140	−0.047	0.141	0.131	−0.115	1.0	0.488*	0.089	0.106	−0.052	0.094	−0.045	0.108	0.159
**BPRS positive symptoms**	−0.075	0.047	0.097	−0.077	0.147	0.078	−0.017	0.488*	1.0	0.008	−0.034	−0.075	0.060	−0.139	0.016	0.101
**BPRS negative symptoms**	−0.119	0.045	−0.026	−0.106	−0.035	−0.029	0.121	0.089	0.008	1.0	0.166	0.129	0.428*	0.412 *	0.406 *	0.011
**BPRS psychological discomfort**	−0.102	0.102	0.016	−0.133	0.060	0.019	0.151	0.106	−0.034	0.166	1.0	0.209	0.383 *	0.399 *	0.407 *	−0.102
**BPRS resistance**	−0.122	0.154	−0.120	−0.129	0.111	−0.126	0.138	−0.052	−0.075	0.129	0.209	1.0	0.311 *	0.317 *	0.338 *	−0.121
**JSQLS dysfunction of psycho-social activity**	−0.140	0.096	−0.134	−0.160	0.100	−0.117	0.121	0.094	0.060	0.428*	0.383 *	0.311 *	1.0	0.250	0.208	0.318 *
**JSQLS dysfunction of motivation and energy**	−0.127	−0.014	−0.009	−0.082	−0.101	−0.016	0.137	−0.045	−0.139	0.412 *	0.399 *	0.317 *	0.250	1.0	0.165	−0.176
**JSQLS level of symptoms and side-effect**	−0.105	0.139	−0.072	−0.117	0.048	−0.054	0.140	0.108	0.016	0.406 *	0.407 *	0.338 *	0.208	0.165	1.0	−0.067
**DIEPSS**	0.104	−0.042	0.127	0.110	0.003	−0.021	−0.140	0.159	0.101	0.011	−0.102	−0.121	0.318 *	−0.176	−0.067	1.0

* Correlation is significant at the 1% level (two-sided).

BPRS, Brief Psychiatry Rating Scale; DIEPSS, The Drug-Induced Extrapyramidal Symptoms Scale; HDS-R, Revised Hasegawa's dementia scale; JSQLS, the Schizophrenia Quality of Life Scale, a Japanese version.

**Table 5:** Relationship among clinical variables and psychometric test scores in groups receiving atypical antipsychotics (correlation coefficient).

Variables assessed	Age	Onset age	Duration of disease	Duration of hospitalization	Years of education	Duration of the antipsychotic medication	HDS-R	BPRS total	BPRS positive symptoms	BPRS negative symptoms	BPRS psychological discomfort	BPRS resistance	JSQLS dysfunction of psycho- social activity	JSQLS dysfunction of motivation and energy	JSQLS level of symptoms and side-effect	DIEPSS
**Age**	1.0	0.158	0.354 *	0.288 *	−0.104	0.259 *	−0.118	−0.120	−0.158	−0.152	−0.126	−0.154	−0.104	−0.130	−0.157	0.152
**Onset age**	0.158	1.0	−0.138	−0.154	−0.062	−0.151	0.156	0.136	0.146	0.113	0.148	0.156	0.128	0.059	0.129	−0.136
**Duration of disease**	0.354 *	−0.138	1.0	0.321 *	−0.057	0.529 *	−0.146	−0.157	−0.154	−0.137	−0.119	−0.142	−0.136	−0.100	−0.158	0.033
**Duration of hospitalization**	0.288 *	−0.154	0.321 *	1.0	−0.037	0.424 *	0.146	−0.045	−0.005	0.078	−0.059	−0.041	0.030	0.012	0.046	0.083
**Years of education**	−0.104	−0.062	−0.057	−0.037	1.0	−0.062	0.137	0.128	0.152	−0.089	0.088	0.137	0.074	−0.097	0.058	0.030
**Duration of the antipsychotic medication**	0.259 *	−0.151	0.529 *	0.424 *	−0.062	1.0	−0.118	0.120	0.062	−0.048	0.039	−0.145	−0.102	−0.068	−0.041	−0.035
**HDS-R**	−0.118	0.156	−0.146	0.146	0.137	−0.118	1.0	0.017	−0.053	0.150	0.158	0.153	0.158	0.157	0.131	−0.150
**BPRS total**	−0.120	0.136	−0.157	−0.045	0.128	0.120	0.017	1.0	0.445 *	0.150	0.096	0.157	0.112	0.084	0.098	0.136
**BPRS positive symptoms**	−0.158	0.146	−0.154	−0.005	0.152	0.062	−0.053	0.445 *	1.0	0.156	0.085	0.134	0.092	0.019	0.144	0.141
**BPRS negative symptoms**	−0.152	0.113	−0.137	0.078	−0.089	−0.048	0.150	0.150	0.156	1.0	0.148	0.109	0.424 *	0.345 *	0.359 *	0.093
**BPRS psychological discomfort**	−0.126	0.148	−0.119	−0.059	0.088	0.039	0.158	0.096	0.085	0.148	1.0	0.185	0.416 *	0.322 *	0.381 *	0.008
**BPRS resistance**	−0.154	0.156	−0.142	−0.041	0.137	−0.145	0.153	0.157	0.134	0.109	0.185	1.0	0.366 *	0.337 *	0.308 *	−0.121
**JSQLS dysfunction of psycho-social activity**	−0.104	0.128	−0.136	0.030	0.074	−0.102	0.158	0.112	0.092	0.424 *	0.416 *	0.366 *	1.0	0.224	0.271	0.289 *
**JSQLS dysfunction of motivation and energy**	−0.130	0.059	−0.100	0.012	−0.097	−0.068	0.157	0.084	0.019	0.345 *	0.322 *	0.337 *	0.224	1.0	0.156	−0.102
**JSQLS level of symptoms and side-effect**	−0.157	0.129	−0.158	0.046	0.058	−0.041	0.131	0.098	0.144	0.359 *	0.381 *	0.308 *	0.271	0.156	1.0	0.031
**DIEPSS**	0.152	−0.136	0.033	0.083	0.030	−0.035	−0.150	0.136	0.141	0.093	0.008	−0.121	0.289 *	−0.102	0.031	1.0

* Correlation is significant at the 1% level (two-sided).

BPRS, Brief Psychiatry Rating Scale; DIEPSS, The Drug-Induced Extrapyramidal Symptoms Scale; HDS-R, Revised Hasegawa's dementia scale; JSQLS, the Schizophrenia Quality of Life Scale, a Japanese version.

We compared each score of the psychometric tests between groups receiving typical and atypical antipsychotics. No significant differences in total HDS-R score were seen between groups. The total BPRS scores, BPRS subscale score for positive symptoms, the JSQLS subscale score for dysfunction of psycho-social activity, and the DIEPSS score were significantly higher in patients receiving typical antipsychotics than in patients receiving atypical antipsychotics (Table 6).

**Table 6:** Comparison of each scale between patients receiving typical and atypical antipsychotics.

	Typical antipsychotic only group	Atypical antipsychotic only group	Between group p value
**HDS-R total score**	19.9±4.6	21.6±6.8	0.101
**BPRS total score**	29.3±16.1	20.8±10.4	<0.001 *
**BPRS positive symptoms**	12.3±6.7	7.9±4.1	0.001 *
**BPRS negative symptoms**	4.7±3.3	5.1±3.6	0.940
**BPRS psychological discomfort**	5.5±3.5	5.1±3.1	0.894
**BPRS resistance**	2.6±1.5	2.9±1.6	0.964
**JSQLS dysfunction of psycho-social activity**	36.6±19.1	25.9±12.7	0.008 *
**JSQLS dysfunction of motivation and energy**	35.2±17.5	38.0±18.8	0.535
**JSQLS level of symptoms and side-effect**	26.3±18.3	24.0±15.7	0.602
**DIEPSS**	5.4±2.1	4.6±2.0	0.009 *

Scores on evaluation scales are mean values (standard deviation).

Student's *t*-test.

* Correlation is significant at the 1% level (two sided).

BPRS, Brief Psychiatry Rating Scale; DIEPSS, The Drug-Induced Extrapyramidal Symptoms Scale; HDS-R, Revised Hasegawa's dementia scale; JSQLS, the Schizophrenia Quality of Life Scale, a Japanese version.

## Discussion

This study was designed to investigate the relationship between long-term administration of antipsychotics and clinical and psychometric variables for schizophrenia inpatients. A further aim was to elucidate the differential influence of typical and atypical antipsychotics on QOL or other symptoms among long-stay inpatients.

The BPRS scores were correlated with the CPZ-equivalent doses of typical antipsychotics and atypical antipsychotics in this study. These were positive significant correlations among the BPRS scores for positive symptoms and the doses of typical and atypical antipsychotics. In these correlations, a high dose of antipsychotics seems to reflect positive symptoms. On the other hand, these correlations might imply that patients stabilized at a lower dose of antipsychotic medication are more likely to have fewer symptoms than those who are receiving a higher dose of medication. However, because this study was cross-sectional, causality of relationships between positive symptoms and the doses of typical and atypical antipsychotics could not be determined.

Significant differences were observed in BPRS subscales for positive symptoms between patients receiving typical and atypical antipsychotics. Psychiatric symptoms generally tended to be more intense among patients who received typical antipsychotics compared with those who received atypical antipsychotics. One possible reason for this finding may be the tendency to continue prescribing typical antipsychotics rather than switching to atypical antipsychotics when psychiatric symptoms persist [4].

A significant positive correlation was found between the subscale score of JSQLS for dysfunction of psycho-social activity and CPZ-equivalent doses of typical but not atypical antipsychotics. Examination of antipsychotic agents and QOL showed that chronic administration of antipsychotic agents increased the levels of feelings of difficulty mixing in social situations, and feeling worried about the future (measured by the JSQLS subscale for the dysfunction of psycho-social activity) in a dose-dependent manner. In regard to the association of typical antipsychotics with the subscale score of JSQLS for dysfunction of psycho-social activity, we viewed the association of typical antipsychotics with extrapyramidal symptoms. The present results suggest that the DIEPSS total score is positively correlated with the CPZ-equivalent doses of typical antipsychotics and that there were significant differences in the DIEPSS score between patients treated with typical antipsychotics and atypical antipsychotics. Furthermore, the DIEPSS score is positively correlated with the JSQLS subscale for dysfunction of psycho-social activity (Table 4, 5). In addition, Crossley *et al.* reported that patients receiving typical antipsychotics experienced more extrapyramidal side effects than patients receiving atypical antipsychotics [34]. The influence of extrapyramidal adverse effects on QOL has already been documented. Ritsner *et al.* used the Montgomery-Asberg Depression Rating Scale (MADRS), the Talbieh Brief Distress Inventory (TBDI), the Abnormal Involuntary Movement Scale (AIMS), and the Quality of Life Enjoyment and Satisfaction Questionnaire in schizophrenia patients and reported that the depression score on the TBDI and the score on the AIMS were predictors of poor QOL [35]. Awad *et al.* used the PANSS, the Hillside Akathisia scale, and the Drug Attitude Inventory to show that subjective QOL is greatly influenced by psychopathology, akathisia, and patients' subjective tolerance of medications, and concluded that effort should be directed toward effective control of psychotic symptoms and minimizing the side effects of antipsychotic drugs to improve the QOL of patients with schizophrenia [24]. Therefore, we thought that patients with extrapyramidal symptoms induced by typical antipsychotics have more subjective discomfort with respect to their symptoms and side effects than patients receiving atypical antipsychotics.

Though this study has something to add about partial correlation, it is assumed that there would be significant relationships among the variables, like what was stated for JSQLS and DIEPPS. Therefore, correlation was performed to investigate the relationship among clinical variables (age, age at disease onset, duration of disease, duration of hospitalization, years of education, and duration of antipsychotic medication) and psychometric test scores. It was evident that there were significant positive correlations among duration of disease, duration of hospitalization, and duration of the antipsychotic medication. On the other hand, there were significant positive correlations between the DIEPSS score and the JSQLS subscale for dysfunction of psycho-social activity, and between BPRS negative symptoms or psychological discomfort or resistance and all JSQLS subscales, respectively (Table 4, 5). This would justify partial correlations that were performed to investigate the relationship between the CPZ-equivalent dose and each psychometric test scores. Despite the relationships between DIEPPS and JSQLS subscale for dysfunction of psycho-social activity in patients, it is suggested that the partial correlation showed that controlling for DIEPPS did not lower the strength of the relationship between typical antipsychotic dose and JSQLS subscale for dysfunction of psycho-social activity. In the same way, despite the relationships between BPRS and JSQLS in patients, the partial correlation showed that controlling for BPRS did not lower the strength of the relationship between typical antipsychotic dose and JSQLS. That is, the relationship between typical antipsychotic dose and JSQLS is not due to patients' experience of extrapyramidal adverse effects and symptoms.

In the present study, partial correlation analysis was applied to indicate the CPZ-equivalent doses of typical and atypical antipsychotics when duration of the antipsychotic medication was partialled out. Ritsner *et al.* [26] reported that the longer the antipsychotic treatment, the better the QOL outcomes. Therefore, we viewed the difference of duration of the antipsychotic medication between the group receiving typical antipsychotics only and the group receiving atypical antipsychotics only. There was no significant difference between the group receiving typical antipsychotics only and the group receiving atypical antipsychotics only. Furthermore, there were no differences in the results when duration of antipsychotic medication was partialled out. Therefore, we could exclude the influence of duration of the antipsychotic medication on the poorer outcome of groups treated with typical antipsychotic medication.

Moreover, Ritsner *et al.* [26] also reported that duration of treatment is a strong factor that may reveal different QOL outcomes for patients receiving atypical versus typical antipsychotics. In the present study, the JSQLS subscale score for dysfunction of psycho-social activity was significantly higher in patients receiving typical antipsychotics than in patients receiving atypical antipsychotics. The above-mentioned results are similar to their results. In our study, it can be suggested that a difference in receiving atypical versus typical antipsychotics influenced QOL without influencing the duration of treatment.

In terms of the relation of frequent relapses and the impact of acute deterioration of symptoms at relapse on social function, there were no differences in the number of two or more hospitalizations between the typical antipsychotic only group and the atypical antipsychotic only group. In addition, the minimum duration of hospitalization was 2.0 years in the typical antipsychotic only group, and 1.8 years in the atypical antipsychotic only group. Because hospitalization data suggest that all patients had a long hospital stay, it is suggested that acute deterioration of symptoms seen within several days of the beginning of hospitalization did not influence our study results.

Ritsner *et al.* [26] found that QOL outcomes were not related to age and education. In the present study, there were no significant differences in these variables between the group receiving typical antipsychotics only and the group receiving atypical antipsychotics only. Furthermore, there were no differences in the results when age and education were partialled out.

With regard to the relationship between atypical antipsychotics and cognitive function, Keefe *et al.* [36] showed that atypical antipsychotics significantly improved cognitive dysfunction in seven of eight categories (attention, executive function, working memory, learning and memory, visuospatial analysis, verbal fluency, digit-symbol substitution, and fine motor function) compared with typical antipsychotics. However, as shown in Table 6, no significant difference in the scores of cognitive function by HDS-R was observed between patients receiving typical and atypical antipsychotics in our study. HDS-R may not have had sufficient sensitivity to detect subtle differences in cognitive functioning between the two antipsychotic-treated groups. In regard to the limitation on clinical assessment of cognitive function in our study, Keefe *et al.* suggested that clinical assessment of cognitive deficits on the Mini Mental Status Examination (MMSE) using the same items as the HDS-R is not a viable alternative to neuropsychological testing to obtain information about cognitive functioning in schizophrenia [37]. Their findings limit the interpretation of the present results. To elucidate the influence of cognitive dysfunction on QOL, further studies using neuropsychological tests such as the Brief Assessment of Cognition in Schizophrenia [38] are necessary.

There is a major limitation to the current study: due to the naturalistic design, drug administration was not controlled before the study, and therefore there were no baseline data at the time of treatment assignment. Although the current results are statistically robust, they should be interpreted with caution, as only an association and not causality can be inferred. At least in part, these limitations have been resolved during the current 6-month follow-up stage of the study. In addition, this study should be interpreted with caution due to certain methodological limitations. First, because the study was cross-sectional, causality of relationships among clinical variables could not be determined. Second, we statistically assessed multiple evaluation items between the group receiving typical antipsychotics only and the group receiving atypical antipsychotics only. However, multiple comparisons were not conducted in our study because each evaluation item was examined individually in this study. Further studies should take these factors into account to determine any statistical differences between two or more groups while evaluating multiple items. With these limitations in mind, this study provides evidence to support the hypothesis that long-term administration of typical antipsychotics has unfavorable associations with psychiatric symptoms, QOL, and drug-induced extrapyramidal symptoms in patients with chronic schizophrenia.

In summary, as shown in Tables 2 and 3, involvement of typical antipsychotics, but not atypical antipsychotics, was specific to the JSQLS subscale score for dysfunction in psycho-social activity. In this cross-sectional study, typical antipsychotics appear to have a stronger association with negative QOL than atypical antipsychotics. In particular, feelings of difficulties in social situations and feeling worried about the future are related to the JSQLS subscale for the dysfunction of psycho-social activity. Therefore, it could be suggested that chronic administration of typical antipsychotics had an unfavorable impact on feelings of difficulties in social situations and feeling worried about the future among patients with schizophrenia. Furthermore, chronic administration of typical antipsychotics induces more side effects that include extrapyramidal symptoms. Based on the results of the present study, the necessity to consider avoiding chronic administration of typical antipsychotics and promptly reducing their doses can be emphasized. We hope that reports on the risks associated with chronic administration of typical antipsychotics, which urge clinicians to switch from typical antipsychotics to monotherapy with atypical antipsychotics, will continue to accumulate.
